# Profile of Some Trace Elements in the Liver of Camels, Sheep, and Goats in the Sudan

**DOI:** 10.1155/2013/736497

**Published:** 2013-12-07

**Authors:** Ibrahim Abdullah Ibrahim, Ali Mahmoud Shamat, Mohammed Osman Hussien, Abdel Rahim Mohammed El Hussein

**Affiliations:** ^1^Central Laboratory, Ministry of Science and Technology, P.O. Box 7099, Khartoum, Sudan; ^2^Institute of Veterinary Research, Animal Resources Research Corporation, P.O. Box 8067, Khartoum, Sudan; ^3^Animal Resources Research Corporation, Al-Amarat P.O. Box 8067, Khartoum, Sudan

## Abstract

One hundred camels (*Camelus dromedaries*) and fifty sheep and goats being adult, male, and apparently healthy field animals were studied to provide data regarding the normal values of some hepatic trace elements. Liver samples were collected during postmortem examination, digested, and analyzed for Cu, Zn, Fe, Co, and Mn using atomic absorption spectrophotometry. The results showed that the differences in mean liver concentrations of Cu, Zn, Fe, and Co between camels, sheep, and goats were statistically significant (*P* < 0.05). Hepatic Cu, Fe, and Co concentrations were higher in camels than in sheep and goats. All liver samples were adequate for Fe and Co, whereas only camel liver was adequate for Cu. In camels, hepatic Zn concentration was inadequately lower than that in sheep and goats. No difference in Mn concentration was detected between camels, sheep, and goats. All liver samples were inadequate compared to free-ranging herbivores. In camels, significant correlation (*r* ^2^ = −0.207, *P* value = 0.04) was detected between Zn and Co, whereas in sheep significant correlation (*r* ^2^ = −0.444, *P* value = 0.026) was detected between Zn and Mn. No significant correlation between trace elements was detected in goats.

## 1. Introduction

Trace elements are involved as essential parts of many physiological activities such as energy production, enzyme activity, hormone production, collagen formation, vitamin and tissue synthesis, oxygen transport, and other physiological processes related to health growth and reproduction and their deficiency leads to wide variety of pathological consequences such as cardiac conditions in addition to immunological and hormonal dysfunctions and metabolic defects [1]. Trace elements deficiencies have negative impacts on the reproductive efficiency of farm animals. The importance of trace mineral nutrition has been recognized for quite some time; however, recent advances in understanding factors influencing trace mineral requirements and their supplementation potential benefits upon health and productivity are proposed by [2–4]. Camels, sheep, and goats are virtually the main source of subsistence for most of the people inhabiting the environmental region loosely referred to as arid and semiarid lands (ASAL) of the Sudan. A major constraint to animal production is the occasional drought; the long dry season, absence of legumes in natural pasture and rapid decline of forage quality of native grasses as the rainy season progresses, and high prevalence of endemic and metabolic diseases are factors contributing directly to low animal production. The traditionally raised livestock do not usually receive mineral supplementation except for common salt (sodium chloride) and natroun (sodium carbonate and sodium bicarbonate) depending almost exclusively upon forage for their mineral requirements. The most common reason to assess the trace mineral status is performance being below expectation. According to McDowell [5] and Kincaid [6] the liver is particularly useful and most accurate for evaluating the animal's status in relation to cobalt, copper, manganese, and selenium. Liver samples from abattoirs have been used in the Sudan to detect areas of copper deficiencies [7] and those from dead animals in some investigations in South Africa [8]. Camel hepatic copper concentration of 155 ppm (range 30–286 ppm) on DM basis was reported by Khalifa et al. [9] in adult Egyptian camels. 274.8 (168–350) and 163.6 (30–543.1) ppm were reported by Tartour [7, 10] in western and eastern Sudan camels, respectively. Abu Damir et al. [11] reported 174.3 (range 22.75–437.5 ppm) in eastern Sudan. Bakhiet et al. [12] reported 103 ± 12.3 ppm and found that hepatic Cu concentration was significantly higher in camels than in cattle, sheep, and goats. Hepatic zinc concentration of 143 ppm and 138.6 ppm was reported by Awad and Berschneider [13] and Abu Damir et al. [11], respectively. The latter found that Zn concentration in camel liver was not different from that in sheep, goats, and cattle. Hepatic iron level in normal camels ranged between 260 and 280 ppm as reported by Abu Damir et al. [14], Awad and Berschneider [13], and Wensvoort [15] but Bakhiet et al. [12] reported 560 ± 38 ppm. Low hepatic manganese values as for other ruminants were reported in the camel; these values ranged between 2 and 10 ppm [11, 13]. The mineral most likely deficient for grazing animals in the world is phosphorus followed by cobalt and copper; no data are available regarding camel blood or hepatic cobalt. The need of ruminants for Co is related to its being an essential element for the synthesis of vitamin B_12_ (cyanocobalamin) in the rumen when the concentration of Co in the rumen fluid falls below a critical level placed at 5 *μ*g/mL [16]; the rate of vitamin B_12_ synthesis by the rumen organisms is reduced below the sheep's needs. Cobalt deficiency signs are not specific and it is often difficult to distinguish between Co deficiency and malnutrition due to low intake of calories and protein.

This research was conducted to quantify and compare the liver trace elements level in camels, sheep, and goats with the aim of identifying potential interventions.

## 2. Materials and Methods

### 2.1. The Study Area

**Figure 1:**
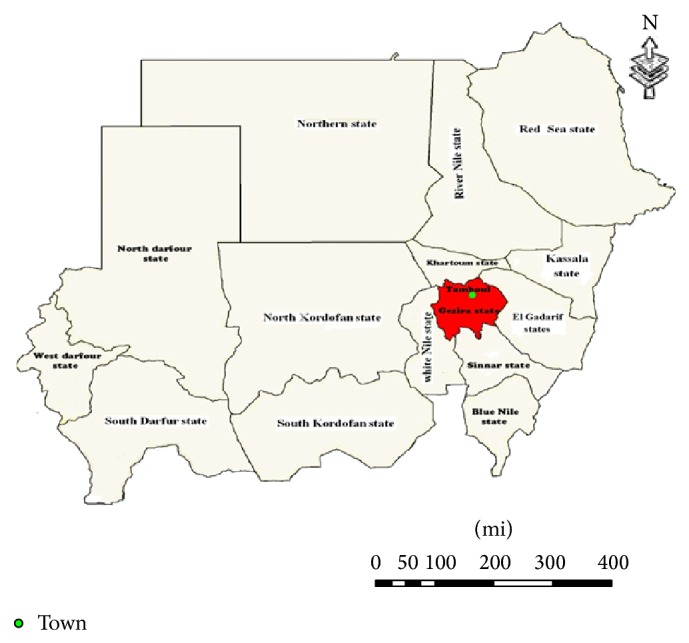
Map of the Sudan showing Gezira state and Tampool area (red colour) where liver samples were collected.

The study area, Al Butana, is part of the central rain lands that provides good grazing for camels, sheep, goats, and cattle stretches from the Ethiopian border in the east to Gezira state in the west roughly occupying the area between isohyets 400 and 700 mm. It comprises 120,000 square kilometers and lies between latitude 13.5°–17.5° N and longitude 32.4°–36.0° E. It is situated in the rich savanna environment (Figure 1).

### 2.2. Collection of Liver Samples

This study was carried out in compliance with the animal welfare code of the Sudan. A total of one hundred fifty liver samples (100 camels (Bushari), 25 sheep (Musalami), and 25 goats (Nubian)) from adult, male, apparently healthy animals were collected from Tambool slaughterhouse, Gezira state, from June 2011 to February 2012. All animals were grazing in the same area. To avoid contamination, stainless surgical blades were used to obtain a small portion of the *lobus quadratus* which was scraped; then an approximately 50 g sample of liver was extracted. The samples were transferred into clean sterile containers and immediately frozen at −20°C until analyzed.

### 2.3. Analysis of Liver Samples

Liver minerals are determined by an atomic absorption spectrophotometer (AAS) (Model A Analyst 700, Perkin-Elmer Corporation, USA) which is equipped with deuterium background corrector. For flame measurements, a 10 cm long slot-burner head, a lamp, and an air-acetylene flame were used. The operating conditions for working elements were set according to manufacturer's instructions. Liver organic matter was destroyed by a wet-oxidation procedure. 5 g of liver sample was placed in a 250 mL Kjeldahl flask with 10 mL of HNO_3_ and 5 mL of H_2_SO_4_ was added to complete digestion. Finally the digest was completed to 100 mL. The standards have the same acid concentration as samples. Liver was analyzed for iron, cobalt, copper, manganese, and zinc by flame AAS equipped with D_2_ corrector. The trace elements concentration was determined after repeatability of assay from the linear standard curve and then multiplied by the dilution factor. The detection limit of the assay ranged from 0 to 6 ppm and the correlation coefficient was 1.

### 2.4. Statistical Analysis

Data collected were subjected to statistical analysis using SPSS version 13 and were expressed as mean ± SE. Data were normally distributed. One way ANOVA test was used for analysis to handle unequal sample size. Correlation between trace elements in each species was done using Pearson's correlation test.

## 3. Results

The mean ± SE concentration values of Cu, Zn, Fe, Co, and Mn in liver of camels, sheep, and goats are shown in Table 1. Hepatic Cu, Fe, and Co concentrations were higher in camels than in sheep and goats. All liver samples were screened against the Cu critical level of <75 ppm [17] and only camel livers were adequate. Regarding hepatic Co and Fe all liver samples were screened against the critical level of <0.05 ppm and that of <180 ppm as suggested by McDowell et al. [18] and McDowell et al. [17], respectively. All livers were found adequate. In camels, hepatic Zn concentration was lower than in sheep and that in goats was also lower than the critical level of <84 ppm as suggested by Miller et al. [19]. No difference in Mn concentration was detected between camels, sheep, and goats. All liver samples were less than the critical level of <8 ppm [20]. The differences in mean liver concentrations of Cu, Zn, Fe, and Co between the three species were statistically significant (*P* < 0.05). In camels, significant correlation (*r* ^2^ = −0.207, *P* value = 0.04) was detected between Zn and Co, whereas in sheep significant correlation (*r* ^2^ = −0.444, *P* value = 0.026) was detected between Zn and Mn. No significant correlation between trace elements was detected in goats.

## 4. Discussion

The chemical composition of body tissues generally reflects the dietary status of domestic and wild animals to varying degrees of accuracy depending on the tissue and the element. Mineral assays on tissues can therefore be used to assist in the detection and definition of a range of mineral inadequacies and excesses in animals. Critical animal tissue concentration was considered to be below or above values associated with specific signs as reported in the literature. In the current study, the mean hepatic concentration of Cu in camels resembles the previous reported values [7, 10–12, 15]. Liver concentrations of Cu, Zn, Fe, and Co obtained from sheep and goats fall within the lower range reported for ruminants [11, 12]. With the exception of Mn, significant differences (*P* < 0.05) were detected in hepatic trace elements between camels, sheep, and goats. Cu concentration in camel liver in the present study was high. This could be attributed to the fact that camels unlike sheep and goats graze more forage trees than grasses which are generally richer in copper and this will lead to accumulation of Cu in the liver [7]. Hepatic Zn concentration in camels was low compared to sheep and goats. This is in agreement with Abu Damir et al. [11] who found lower hepatic Zn values (39.6 ± 17.7 ppm) in Sudanese camels compared to other livestock. Bakhiet et al. [12] also reported lower hepatic Zn concentration (34.7 ± 1.02 ppm) in Sudanese camels compared to sheep and goats. Higher hepatic Fe concentration in camels noticed in this study (545.9 ± 27.9) typifies the results recorded by Tartour [10] and Bakhiet et al. [12]. However, in natural conditions, iron deficiency is not observed in ruminants [21]. As for other ruminants, hepatic Co concentrations are generally low in camels compared to other trace elements according to different authors [11, 12, 15], a fact that was well documented in the current study. This could be due to the fact that ruminants need Co as an essential element for the synthesis of vitamin B_12_ in rumen.

## 5. Conclusion

The results of these trace elements reported in the present study are considered a good source of information for healthcare people concerned about any disorder linked to deficiency of these elements. It is recommended that further studies should be done to determine the profile of trace elements in animals at slaughter in different regions of the Sudan to monitor the possible risk of livestock trace elements poisoning/deficiency by determining the highest/lowest levels of minerals detected in animal tissue.

## Figures and Tables

**Table 1:** Mean ± SE concentrations of Cu, Zn, Fe, Co, and Mn in the liver of camels (*n* = 100), sheep (*n* = 25), and goats (*n* = 25) in Tambool slaughterhouse, Gezira state, from July 2011 to February 2012.

Parameter	Camels	Sheep	Goats	*P* value
Cu (ppm)	100.7 ± 5.2	72.1 ± 4.2	51.4 ± 2.1	*P* < 0.05
Zn (ppm)	32.9 ± 2.3	141.9 ± 3.9	134.8 ± 2.9	*P* < 0.05
Fe (ppm)	545.9 ± 27.9	222.7 ± 3.8	202.8 ± 3.4	*P* < 0.05
Co (ppm)	1.87 ± 0.35	0.46 ± 0.22	0.30 ± 0.17	*P* < 0.05
Mn (ppm)	6.9 ± 0.86	6.3 ± 8.3	6.5 ± 1.4	*P* > 0.05
